# High-resolution array CGH clarifies events occurring on 8p in carcinogenesis

**DOI:** 10.1186/1471-2407-8-288

**Published:** 2008-10-07

**Authors:** Susanna L Cooke, Jessica CM Pole, Suet-Feung Chin, Ian O Ellis, Carlos Caldas, Paul AW Edwards

**Affiliations:** 1 Department of Pathology and Hutchison/MRC Research Centre, University of Cambridge, Cambridge, UK; 2 CRUK Cambridge Research Institute and Department of Oncology, University of Cambridge, Cambridge, UK; 3 Department of Histopathology, Nottingham City Hospital NHS Trust and University of Nottingham, Nottingham, UK

## Abstract

**Background:**

Rearrangement of the short arm of chromosome 8 (8p) is very common in epithelial cancers such as breast cancer. Usually there is an unbalanced translocation breakpoint in 8p12 (29.7 Mb – 38.5 Mb) with loss of distal 8p, sometimes with proximal amplification of 8p11-12. Rearrangements in 8p11-12 have been investigated using high-resolution array CGH, but the first 30 Mb of 8p are less well characterised, although this region contains several proposed tumour suppressor genes.

**Methods:**

We analysed the whole of 8p by array CGH at tiling-path BAC resolution in 32 breast and six pancreatic cancer cell lines. Regions of recurrent rearrangement distal to 8p12 were further characterised, using regional fosmid arrays. FISH, and quantitative RT-PCR on over 60 breast tumours validated the existence of similar events in primary material.

**Results:**

We confirmed that 8p is usually lost up to at least 30 Mb, but a few lines showed focal loss or copy number steps within this region. Three regions showed rearrangements common to at least two cases: two regions of recurrent loss and one region of amplification. Loss within 8p23.3 (0 Mb – 2.2 Mb) was found in six cell lines. Of the genes always affected, *ARHGEF10 *showed a point mutation of the remaining normal copies in the DU4475 cell line. Deletions within 12.7 Mb – 19.1 Mb in 8p22, in two cases, affected *TUSC3*. A novel amplicon was found within 8p21.3 (19.1 Mb – 23.4 Mb) in two lines and one of 98 tumours.

**Conclusion:**

The pattern of rearrangements seen on 8p may be a consequence of the high density of potential targets on this chromosome arm, and *ARHGEF10 *may be a new candidate tumour suppressor gene.

## Background

Rearrangements of the short arm of chromosome 8 (8p) are one of the most common genetic events in breast [1-3], pancreatic [4] and many other epithelial carcinomas [5]. Although these rearrangements have been studied using a variety of techniques, including cytogenetics, CGH (comparative genomic hybridisation), array CGH and LOH (loss of heterozygosity) analysis [1-4], their resolution, with the exception of studies on 8p12 which are discussed below, has been insufficient to provide a feasibly small number of candidate genes for further analysis.

Loss of heterozygosity (LOH) studies suggest that there are at least three tumour suppressor genes on 8p, in bands 8p23, 8p22, and 8p21, lost in several epithelial tumour types [6-8]. The highest resolution of these studies is just over 1 Mb, showing that bladder tumours have a common region of LOH between microsatellite markers D8S504 (1.0 Mb) and D8S264 (2.1 Mb) in 8p23.3 [8]. In support of this a lower resolution LOH study of ovarian adenocarcinomas identifies D8S264 (2.1 Mb) as showing the second highest rate of LOH on 8p, with the highest rate at D8S1827 (14.9 Mb) in 8p22 [7].

Cytogenetic and array CGH studies suggest that distal 8p is frequently lost through an unbalanced translocation that breaks in 8p11-12 [3,9]. The 8.8 Mb between 29.7 Mb and 38.5 Mb, encompassing 8p12, is the only region of 8p that has been extensively studied at high resolution, using tiling-path BAC array CGH [10,11]. The translocation breakpoints are usually within, or proximal to, the chromosome band 8p12 and several of them are within the *NRG1 *(*neuregulin 1*) gene (32.2 Mb; midpoint on NCBI Build 36) [12,13]. Most recent research has focussed on this breakpoint region and more proximal changes and array CGH has not been applied to the distal candidate regions identified by the earlier LOH studies.

The other known recurrent feature of 8p rearrangements, at least in breast cancer, is amplification of 8p11-12 in 10–25% of cases, correlating with poor prognosis [11,13-15]. This amplicon was originally suggested to target the *FGFR1 *(*Fibroblast Growth Factor Receptor 1*) gene [16], a member of the same family as *FGFR2 *(*Fibroblast Growth Factor Receptor 2*), a known oncogene and driver of amplification. However, it has subsequently been shown that *FGFR1 *is not always included in the minimal region of amplification and other candidate oncogenes have been suggested [11,13-15,17,18]. Detailed analysis of 8p11-12 amplification shows that there may be as many as four sub-regions of amplification [11,17], raising the possibility that there are multiple cancer-relevant genes in 8p11-12.

Array CGH studies at around 1 Mb resolution of breast [19] and pancreatic cancer [20] confirm the general pattern of loss of 8p but are of insufficient resolution to study individual changes and identify candidate target genes. Two tiling-path array CGH studies have been carried out on seven [21] and ten [22] breast cancer cell lines. The first mentions two changes on 8p [21]. MCF-7 has lost the whole of 8p and T47D has two small deletions in 8p23.1, although we now suggest that this is due to normal copy number variation (see results). At least two further changes – breaks at 8p12 with distal loss – are visible on the ZR-75-30 and T47D karyograms included in the supplementary data, but the raw data is not available for further analysis. The second study identifies amplification of 8p11-12 in JIMT-1 [22].

Overall the picture of events occurring on 8p, and especially distal 8p, is rather confused, due at least in part to the lack of high-resolution data on 8p rearrangements and the lack of array CGH data for the regions highlighted by LOH as likely to contain tumour suppressor genes. Array CGH suggests that a tumour suppressor gene is located at, or just distal to, the 8p12 breakpoint cluster [10], while LOH analysis suggests the existence of several more distal tumour suppressor genes [6-8]. In spite of recent analyses that might suggest a relatively small overlap in alterations across different tumour types [23], the general pattern of changes on 8p is similar across several tumour types and is therefore potentially driven by the same selective pressures.

Our objectives were to investigate whether there were recurrent events on 8p outside the well-studied 8p11-12 region; in particular to look for deletions in distal regions, previously suggested by LOH to harbour a tumour suppressor gene; and to try and make sense of the overall pattern of changes seen on 8p. We set out to analyse the whole of 8p by tiling-path-resolution array CGH in a panel of breast cancer cell lines, and to investigate further regions of recurrent rearrangement. We had previously obtained SKY karyotypes for 27 of the 32 breast cancer cell lines used in this study. Additionally we included six pancreatic cancer cell lines that show rearrangements of 8p at cytogenetic resolution .

## Methods

### Cell lines and primary tumours

Details of cancer cell lines can be found in Additional file 1. Immortalised human breast luminal epithelial line HB4a [24] was obtained from Professor Mike O'Hare (LICR/UCL Breast Cancer Laboratory, University College, London, UK) and normal male lymphoblastoid line m62 obtained from Dr Chris Tyler-Smith (Wellcome Trust Sanger Institute, Hinxton, UK). Both were cultured in DMEM:F12 (Invitrogen, Paisley, UK).

All tumours were invasive breast carcinomas of small size (91% < 3 cm) and low Nottingham Prognostic Index, collected between 1990 and 1996 as part of the Nottingham/Tenovus series [25-29]. 65% were ER positive. The tissue microarray consisted of over 100 breast tumours. Matching RNA, TRIzol^®^-extracted from fresh-frozen tissue, was available for 61 of these tumours.

### Array construction

For the 8p tiling path array, 328 overlapping BAC clones for the length of 8p were selected from the 32 k clone set available from the BACPAC Resource Centre (CHORI, CA, USA) [30]. Additional clones to close gaps were obtained from Invitrogen and the Wellcome Trust Sanger Institute. A subset of 6% of these BACs was sequenced to confirm their identity. All except one matched their expected position on the human genome. The remaining clone showed evidence of containing more than one sequence and was substituted on the final array. The selected overlapping BAC clones for 8p provided an average clone spacing of 138 kb. A spot failure rate of around 10% resulted in an actual resolution averaging 155 kb. For the fosmid array, 123 fosmids were obtained from the Sanger Institute, mainly covering the regions of 8p21.3 (27 clones between 21.59 Mb and 22.25 Mb) and 8p22 (43 clones between 12.97 Mb and 15.68 Mb tiling three genes but not intergenic regions), identified by BAC array CGH as containing multiple rearrangements, to a resolution of 0.04 Mb. BACs were amplified by the method of Fiegler *et al*. [31] and printed with the custom genomic 8p12 array first described in Huang *et al*. [32] on CodeLink slides (Amersham Biosciences, Amersham, UK). Fosmids were prepared by the same method and printed as a separate array.

### Array hybridisation

Array hybridisations were carried out essentially as described in Garcia *et al*. [14] and Pole *et al*. [10]. Tumour genomic DNA was labelled with Cy3-dCTP and a pool of normal female genomic DNA with Cy5-dCTP (Amersham Biosciences) using Bioprime labelling kits (Invitrogen). Samples were hybridized to arrays overnight in the presence of Cot1 DNA (Invitrogen), and washed in PBS/0.05% Tween-20 at room temperature, 50% formamide/SSC at 42°C and then again in PBS/0.05% Tween-20. Arrays were scanned on an Axon 4100A scanner with data collected using GenePix Pro 4.1 software (Axon Instruments, CA, USA) and analyzed in Excel. For array painting, chromosomes (provided by Dr Karen Howarth, University of Cambridge, UK) were sorted by flow-cytometry and amplified by GenomiPhi (GE Healthcare, Buckinghamshire, UK) according to the method of Howarth *et al*. [33]. They were hybridised against GenomiPhi-amplified normal female genomic DNA.

The array performance was tested by self-self and male-female hybridisations and using known 8p rearrangements in T47D (Additional file 2). Self-self hybridisation gave a standard deviation (SD) of ± 0.08 and male-female hybridisation ± 0.04 SD. Log_2 _ratio shifts in response to copy number changes were tested by hybridisation of T47D against normal female gDNA. Copy number changes were estimated by inspection, taking into account both the shift in log_2 _ratio and the level of noise for each sample. In general a change in log_2 _ratio of 0.5 or more was scored as a change in copy number and a gain in log_2 _ratio of 2 or more was scored as amplification.

### Chi-squared test

The probability of the observed distribution of rearrangements on 8p occurring in the absence of any selection or pre-disposition to breakage at certain sites was calculated by chi-squared test. Chromosome 8 was divided into the major bands (8p11, 12, 21, 22, 23) and the observed number of rearrangements in each band tested against the number expected at random according to the size of each band.

### Fluorescence *in situ *hybridization (FISH)

Metaphase preparation and FISH was carried out as described previously [10,34]. Chromosome 8 paint was made using flow sorted chromosomes kindly provided by Patricia O'Brien and Professor Ferguson-Smith (University of Cambridge, UK). All BACs used for FISH were tested individually on normal (m62) metaphases for hybridisation to the correct region of chromosome 8.

### FISH on paraffin embedded tumours

Tissue microarray slides were prepared from formalin-fixed paraffin embedded tissue and analysed according to the method of Chin *et al*. [35]. Probes for FISH on paraffin embedded tumours were prepared in the same way as for conventional FISH. A pool of three BACs, RP11-529P14 (21.8 Mb; positions are given as BAC midpoints according to NCBI Build 36), RP11-67H12 (21.9 Mb) and RP11-70D12 (22.0 Mb), was used for the test region and a pool of two BACs, RP11-381G11 (19.4 Mb) and RP11-222M11 (19.5 Mb), was used as a reference.

### RT-PCR

RNA was reverse transcribed using SuperScript III Reverse Transcriptase (Invitrogen) and oligo dT primer. Quantitative real-time PCR was carried out, in triplicate, in 10 μl reactions containing 1× SYBR Green PCR Master Mix (Applied Biosystems, CA, USA), 2.5 pmol both forward and reverse gene specific primer and 1 μl of 10-fold diluted cDNA. Cycling conditions were 50°C for 2 min, 95°C for 10 min then 40 cycles of 95°C for 15 s, 60°C for 1 min and a final dissociation step of 95°C for 15 s, 60°C for 15 s and 95°C for 15 s in an ABI PRISM 7900 HT Sequence Detection System (Applied Biosystems).

Primer sequences are given in Additional file 3. Standard curves for each primer pair were used to calculate amplification efficiency coefficients and melting curve analysis following qPCR confirmed that each primer amplified a single product. Relative expression levels were calculated as:

$$\frac{{\left(\text{Average Sample Ct for gene }-\text{ Average reference Ct for gene}\right)}^{\text{Amplification Coefficient for gene}}}{{\left(\text{Average Sample Ct for GAPDH }-\text{ Average reference Ct for GAPDH}\right)}^{\text{Amplification Coefficient for GAPDH}}}$$

For primary samples expression levels were compared to the median-expressing tumour. Purified luminal cells, purified basal cells [36] (both kindly provided by Professor Mike O'Hare, UCL, London), a range of commercial normal RNAs (Stratagene (Cambridge, UK), USBiological (Europa Bioproducts Ltd, Wicken, UK), Ambion (Huntingdon, UK), BioChain (AMS Biotechnology, Abingdon, UK), Clontech (Basingstoke, UK)), and normal breast line HB4a were used as normal controls.

## Results

**Figure 1:**
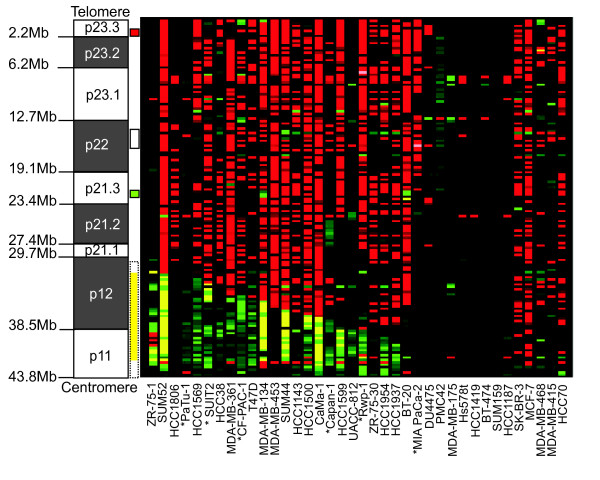
**Array CGH of 8p in 38 cancer cell lines**. The ideogram shows the short arm of chromosome 8 with the position of the chromosome bands indicated on the left and regions of recurrent change represented by coloured boxes on the right of the ideogram. Previously identified positions of recurrent rearrangement (8p11-12) are indicated by a dashed outline. The novel regions identified by this study are indicated by a solid outline. For these summary boxes red indicates a common region of loss in 8p23.3, green a common region of gain/amplification in 8p21.3 and white indicates a region with various rearrangements including gain, loss and breakpoints in 8p22. Array CGH results are displayed as a heat map using CGHAnalyzer (, [64]). The first twenty-four lines show a typical pattern of 8p12 rearrangement with breakpoints, distal loss and occasionally proximal amplification. The next four lines have atypical patterns of 8p rearrangement. The final ten lines show no copy number changes within 8p but the last five have lost the whole of 8p with respect to 8q. Red, loss relative to the ploidy of the cell line; Black, no change; Green, gain; Yellow, high level amplification; Grey, no copy number data; * indicates a pancreatic line.

Array CGH results were obtained for a set of 32 breast and six pancreatic cancer cell lines (Figure 1, Additional files 4). Five cell lines had lost the whole of 8p with respect to 8q, five had no copy number change on 8p, and the remaining 28 had at least one unbalanced rearrangement (Additional file 5). By chi-squared analysis the distribution of breakpoints across the different chromosome bands was non-random (p = 0.0001), suggesting that rearrangements of 8p are under selective pressure or that breaks occur non-randomly due to breakage-prone regions. We identified, in addition to the expected translocation breakpoints and amplicon in 8p11-12 (29.7 Mb – centromere), three further regions of recurrent rearrangement (Figure 1). These are located more distally on 8p: two overlapping amplicons, which defined a possible novel region of amplification in 8p21.3 (19.1 Mb – 23.4 Mb), a region of recurrent rearrangement in 8p22 (12.7 Mb – 19.1 Mb) and a region of focal recurrent loss in 8p23.3 (0 Mb – 2.2 Mb). They identify a limited number of candidate genes in these regions for further study.

Normal copy number polymorphisms were observed at around 7 Mb in 8p23.1, where there is a high level of segmental duplication and a family of homologous and highly polymorphic genes, the *defensins *[37,38]. FISH with BACs RP11-185K20 (7.80 Mb; positions are given as BAC midpoints according to NCBI Build 36) and RP11-43B8 (7.76 Mb) from this region on normal lymphoblastoid interphase nuclei confirmed copy number differences were present between the homologous chromosomes (Additional file 2).

### 8p11-12 amplicon

As we described previously, most of the cell lines (25/28) with rearrangements of 8p had at least one copy number step in 8p11-12, with loss of most or all of 8p distal to this region. Similarly, six out of the eight amplicons found were in the known region of amplification in 8p11-12. A full analysis of these events is given in Pole *et al*. [10].

### A novel amplicon in 8p21.3

**Figure 2:**
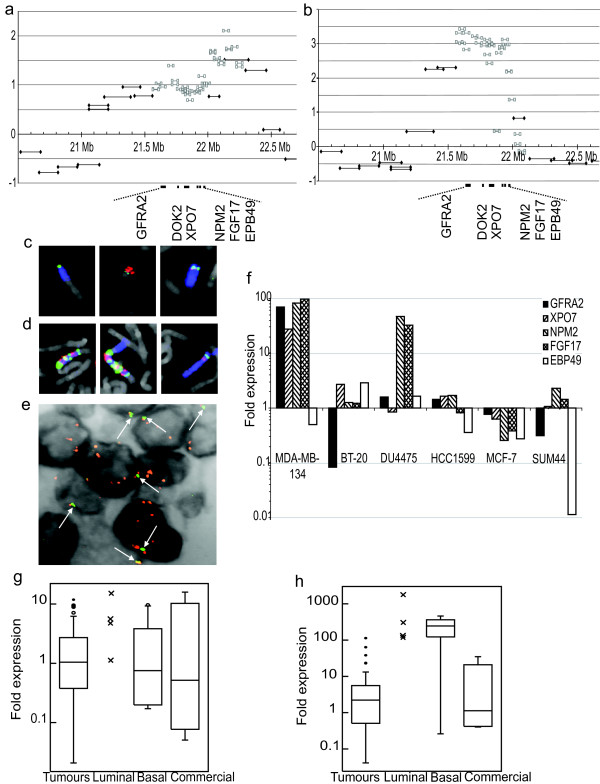
**Analysis of the 8p21.3 amplicon**. **(a) **and **(b) **BAC and fosmid array CGH of the 8p21.3 amplicons in BT-20 and MDA-MB-134 respectively. **(a) **BT-20 showed amplification between BAC clones RP11-419L22 (21.13 Mb; positions are given as midpoints on NCBI Build 36) and RP11-582J16 (22.50 Mb). **(b) **MDA-MB-134 showed amplification between BAC clones RP11-458H21 (21.28 Mb) and RP13-600L4 (22.04 Mb). The distal edges of both amplicons fell in a region containing no known genes but extended proximally into a gene-dense region. Genes within the overlap are marked. Log_2 _ratio of the fluorescence intensity is plotted against position on 8p. Grey squares, fosmids; Black diamonds, BACs. **(c) **FISH of the three chromosomes containing material from the 8p21.3 amplicon in BT-20. Blue, chromosome 8 paint; Green, RP11-459H21 (21.29 Mb); Red, RP11-235B11 (22.38 Mb). **(d) **FISH of the three chromosomes containing material from the 8p21.3 and 8p12 amplicons in MDA-MB-134. Blue, chromosome 8 paint; Green, BAC RP11-135I5 (21.49 Mb); Red, RP11-104D16 (40.25 Mb). **(e) **FISH of the 8p21.3 amplicon on a primary breast tumour paraffin section. Red, BAC pool positioned in the amplicon (centred at 21.9 Mb); Green (also indicated by arrows), BAC pool distal to the amplicon (centred at 19.5 Mb); Grey, inverted DAPI. **(f) **Expression levels of the five genes included in both the BT-20 and MDA-MB-134 amplicons, shown on the y-axis as a log_10 _scale of -fold expression compared to normal breast cell line HB4a and normalised to *GAPDH *as an internal control. Expression levels of **(g) ***FGF17 *and **(h) ***NPM2 *in primary tumours and normal controls. Crosses, purified luminal samples; Open circles, possible outliers (value more than 1.5× inter-quartile range above the third quartile); Filled circles, outliers (value more than 3× inter-quartile range above the third quartile).

Two cell lines, MDA-MB-134 and BT-20, showed amplification in an overlapping region of 8p21.3. This was confirmed and the amplicon further mapped by fosmid array CGH (Figures 2a and 2b, Additional file 6). Both amplicons contained the genes *GFRA2 *(*GDNF Family Receptor Alpha-2*) (21.6 Mb), *DOK2 *(*Docking protein 2*) (21.8 Mb), *XPO7 *(*exportin 7*) (21.9 Mb), *NPM2 *(*nucleophosmin/nucleoplasmin family member 2*) (21.9 Mb) and *FGF17 *(*Fibroblast Growth Factor 17*) (22.0 Mb). The centromeric edge of the amplicon in MDA-MB-134 fell within gene *EPB49 *(*Erythrocyte membrane Protein Band 49*) (22.0 Mb), whereas the amplicon in BT-20 extended proximally to encompass up to a further eighteen genes and one micro RNA. By both fosmid and BAC array CGH, the amplicon in BT-20 consisted of two levels of amplification with a step up at 22.02 Mb (Figure 2a), placing the highest level of BT-20 amplification outside the MDA-MB-134 amplicon.

FISH was used to confirm these amplifications and to investigate their structure. FISH on BT-20 with BAC RP11-235B11 (23.0 Mb), from the region of highest amplification, and BAC RP11-459H21 (21.2 Mb), from the adjacent region of gain, suggested that these two regions of copy number increase were not co-localised (Figure 2c). There was one apparently normal chromosome 8 with signals from both BACs. All extra copies of the region of highest amplification were found on a single small derivative chromosome. One copy of the region of lower amplification was seen on this derivative and one extra copy was present on a separate derivative chromosome 8.

MDA-MB-134 is known to have an 8p12 amplicon that is part of a marker chromosome containing co-amplified 8 and 11 sequences [17]. FISH on MDA-MB-134 was carried out with BAC RP11-135I5 (21.5 Mb), from the 8p21.3 amplicon, and BAC RP11-104D16 (40.2 Mb) from the 8p12 amplicon. This showed that the 8p21.3 amplicon was intermingled with the 8p12 and 11q13 amplicons on the der(?)t(8;11)ins(8;11) (Figure 2d). Incidentally, FISH also showed that the two copies of this marker chromosome are not identical but have undergone further evolution since endoreduplicating. In addition there was one apparently normal copy of chromosome 8.

Dramatic amplification of the region that was both gained in BT-20 and amplified in MDA-MB-134 was found in a single primary breast tumour, out of 98 useable tumour cores, by FISH on a primary breast tumour tissue microarray, with a pool of BACs positioned within the amplicon (21.8 Mb-22.1 Mb) and a pool positioned 2.5 Mb distal to the amplicon (19.3 Mb-19.6 Mb) (Figure 2e).

Expression of the genes within the amplicon, *GFRA2*, *XPO7*, *NPM2*, *FGF17 *and *EPB49*, was analysed to see if amplification had an effect on expression levels. Quantitative RT-PCR was performed on cDNA from the two cell lines that had amplification, and a further four cell lines without amplification in this region (Figure 2f). *DOK2 *was excluded from quantitative expression studies as expression was not detectable by conventional PCR in normal breast line HB4a, BT-20 or MDA-MB-134 (results not shown). MDA-MB-134 showed over-expression of *GFRA2*, *XPO7*, *NPM2 *and *FGF17 *compared to HB4a but 0.5-fold expression of *EPB49*, consistent with the edge of the amplicon falling within this gene (Figure 2f). BT-20 showed marginal, up to 3-fold, over-expression of *XPO7 *and *EPB49*. Interestingly, one cell line, DU4475, showed huge over-expression of *NPM2 *and *FGF17 *in the absence of amplification (Figure 2f).

Since *NPM2 *and *FGF17 *were over-expressed in two cancer cell lines, expression levels were analysed in 61 primary tumours, from the primary breast tumour series analysed by FISH, for which cDNA was available (Figures 2g and 2h). However, cDNA was unavailable for the tumour that showed 8p21.3 amplification by FISH. For both genes, cDNA made from five commercial normal breast RNAs showed extreme variation – over 100-fold difference – in expression across the samples, highlighting the problem of identifying suitable normal controls for primary tumour samples. The variation between commercial normal samples may reflect differences in how they are obtained, for example if RNA is extracted from tissue obtained after reduction mammoplasty it may be more representative of adipocytes than breast epithelium. These technical concerns may limit the utility of commercially available normal RNA as a reference. Purified breast luminal and basal cells [36] were included as further controls and tended to be more consistent in their expression levels.

None of the tumours expressed *FGF17 *above the range of the control groups (Figure 2g). Three tumours expressed *NPM2 *at a higher level than any of the commercial controls, although at a lower level than the normal luminal and basal cells (Figure 2h). Although tumours do not express either gene outside the range of normal controls, there are four outliers/probable outliers for each gene, which have expression at significantly higher levels than the rest of the tumours (Figures 2g and 2h). Due to the variability of the normal controls the most significant observation may be that there were outliers over-expressing both genes within the tumour group.

### 8p22 rearrangements

**Figure 3:**
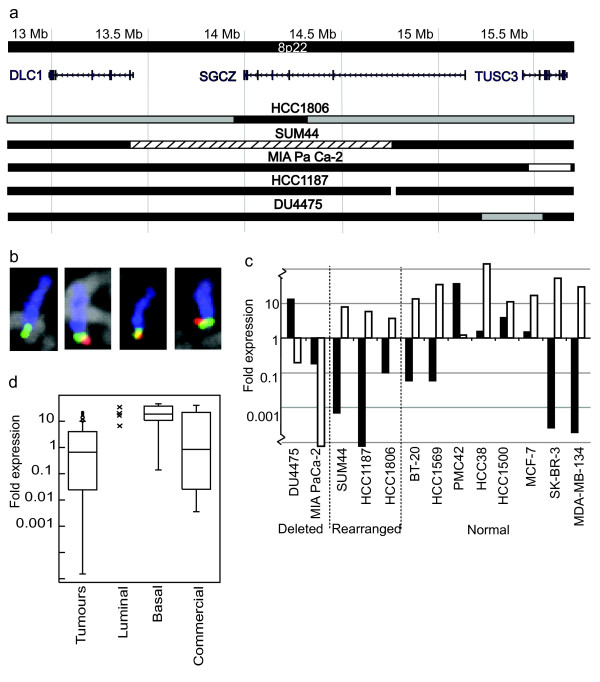
**Analysis of 8p22 rearrangements**. **(a) **Rearrangements between 12.9 Mb and 15.6 Mb in 8p22. Black bar, normal copy number for 8p; Grey, heterozygous loss; White with black outline, homozygous loss; White with no outline, breakpoint of reciprocal translocation; Black hatching, gain. **(b) **FISH showing the deletion of the *TUSC3 *promoter on one of the four copies of chromosome 8 in DU4475. Blue, chromosome 8 paint; Green, RP11-480M18 (15.0 Mb); Red, RP11-314P10 (15.6 Mb). **(c) ***DLC1 *(black bars) and *TUSC3 *(white bars) expression levels relative to normal breast line HB4a. Note *TUSC3 *expression is decreased in MIA PaCa-2 and DU4475, the two cell lines with deletions of this gene. **(d) ***TUSC3 *expression in primary breast tumours and various normal breast samples (see Methods). *TUSC3 *expression is lower in a subset of tumours than in any control group.

A cluster of copy number steps that might affect a common target gene were found between 12.9 Mb and 15.6 Mb in 8p22 (Figure 3a, Additional file 5). Unbalanced changes in this region were seen in four cell lines: HCC1806, SUM44, DU4475, and pancreatic line MIA PaCa-2, which has a homozygous deletion previously reported by Bashyam *et al*. ([4]. In addition, we recently found a reciprocal translocation breakpoint at 14.7 Mb in HCC1187 by array painting, i.e. hybridisation of individual chromosomes to arrays [33] (Figure 3a). These rearrangements were fine-mapped to a resolution of 0.04 Mb, by array CGH on a fosmid array covering the three genes in this region, 13.0 Mb-13.4 Mb (*DLC1*), 14.0 Mb-14.5 Mb (*SGCZ – sarcoglycan zeta*) and 15.4 Mb-15.7 Mb (*TUSC3*) (Figure 3a, Additional file 6).

In SUM44 there were two copy number steps resulting in an extra copy of a retained fragment of the region between 13.6 Mb, within the second intron of *DLC1 *by fosmid array CGH, and 14.6 Mb, within *SGCZ*. DU4475 had a deletion between 15.4 Mb and 15.6 Mb, virtually the same region as the homozygous deletion in MIA PaCa-2. The copy number shift observed in DU4475 was relatively small and FISH, with BAC RP11-314P10 (15.6 Mb) within the deletion and BAC RP11-480M18 (15.0 Mb) just outside it, showed that the deletion was only present on one out of the four copies of chromosome 8 present in DU4475 cells (Figure 3b).

In HCC1806 two copy number steps fell in this region (Figure 3a). Comparison of array painting results [33] and BAC array CGH allowed us to assign these to two separate copies of chromosome 8. There was a small deletion between 12.6 Mb and 13.9 Mb, including all of candidate cancer gene *DLC1*, on an otherwise normal copy of chromosome 8, and a much larger interstitial deletion between 14.2 Mb and 31.5 Mb on a del(8)(8p12-22). Hybridisation of flow-sorted del(8)(8p12-22) to the fosmid array pinpointed the distal edge of the deletion to between 14.19 Mb and 14.24 Mb, within the gene *SGCZ *(Figure 3a).

Overall *DLC1*, the most well characterised tumour suppressor candidate in 8p22, was only affected in a single cell line by a copy number decrease that included two other genes. This result is perhaps not surprising as it is known that *DLC1 *expression does not correlate with copy number [39] and, in several tumour types, down-regulation is caused by promoter methylation [40]. In support of this, *DLC1 *expression was decreased in 62% (8/13) cell lines analysed by quantitative PCR, including four lines without detectable genomic changes in this region (Figure 3c).

In contrast, *TUSC3 *was affected by two small deletions that did not include any other gene. *TUSC3 *showed decreased expression in these two lines with deletions but not in any other line (Figure 3c). Anecdotally, *TUSC3 *also showed decreased expression in a panel of 61 primary breast tumours (Figure 3d). This identified six tumours that had completely lost expression of *TUSC3*, and a further thirteen that expressed it at a lower level than the range observed in normal breast luminal and basal cells.

Neither the 5' nor the 3' end of *SGCZ *showed expression in the lines with copy number changes within it (HCC1187, SUM44), or model normal breast line HB4a (results not shown), so it is neither a likely target of deletion nor of activation or inactivation by the translocation or insertion breakpoints.

### Loss of 8p23.3

**Figure 4:**
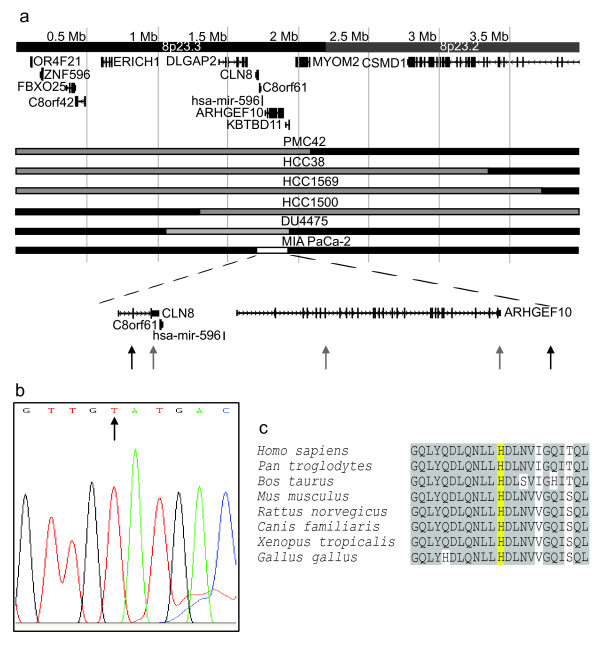
**Analysis of losses in 8p23.3**. **(a) **Losses between 3.7 Mb and the telomere. Black bar, normal copy number for 8p; Grey, heterozygous loss; White with black outline, homozygous loss. Black arrows, position of primer pairs that amplify a product from MIA PaCa-2 genomic DNA i.e. lie outside the homozygous deletion; Grey arrows, position of primer pairs that do not amplify a product from MIA PaCa-2 genomic DNA. **(b) **Sequencing trace showing the point mutation in exon 19 of ARHGEF10. The normal sequence has a cytosine at the position indicated by the arrow. **(c) **Conservation of ARHGEF10. Yellow, the residue that is converted from a histidine to tyrosine by the point mutation in DU4475; Grey, conserved residues.

Six cell lines showed losses close to the telomere, within 8p23, all including a minimum common region of deletion from 1.7 Mb to 1.9 Mb (Figure 4a). PMC42, HCC38 and HCC1569 had losses from the telomere to 2.2 Mb, 3.4 Mb and 3.7 Mb respectively; HCC1500 had a deletion between 1.3 Mb and 36.5 Mb; DU4475 had a heterozygous deletion between 1.1 Mb and 1.9 Mb and MIA PaCa-2 had a homozygous deletion between 1.7 Mb and 1.9 Mb, first reported by Bashyam *et al*. ([4]. In order to identify the genes affected by the minimum common region of loss, the homozygous deletion in MIA PaCa-2 was mapped by PCR. It included exon three, but not exon 2 of *CLN8*, and all of *C8orf61*, *ARHGEF10 *and micro RNA *hsa-mir-596 *(Figure 4a).

Since DU4475 had a heterozygous deletion, in order to fit the classical model of a tumour suppressor gene, we would expect the remaining copy of the target gene to be disrupted by some other mechanism, such as point mutation, in this line. Expression data for multiple cancer cell lines (not shown) suggested *ARHGEF10 *as a more likely target gene than *CLN8*, since ARHGEF10 expression was decreased by more than 50% in 83% (15/18) of lines tested. We therefore sequenced *ARHGEF10 *in DU4475 and found a C → T point mutation in exon 19 (Figure 4b). This causes a non-conservative histidine to tyrosine substitution in the protein product at position 733, a highly conserved region (Figure 4c). Although paired normal DNA is not available for this cell line this nucleotide change is not listed as a known SNP (single nucleotide polymorphism) in the Entrez SNP database (Build 129).

The breast cancer cell line DU4475 was unique in having five separate small deletions on 8p (Additional file 5), at least three of which coincided with candidate tumour suppressor genes: a deletion of the promoter of candidate tumour suppressor *TUSC3 *(*Tumour Suppressor Candidate 3*) (15.6 Mb) in 8p22, an approximately 1 Mb deletion between 21.9 Mb and 23.0 Mb in 8p21, containing candidate cancer genes *RHOBTB2 *(22.9 Mb) and *DR5 *(*Death receptor 5*) (23.0 Mb) [41] but not extending as far as *DR4*, and a deletion in 8p23.3, which emerged as a common region of loss in this study.

## Discussion

This high-resolution array CGH study of 8p in carcinomas has allowed us to build a picture of the more distal events on 8p and to put each change into context. Previous array CGH studies have focused on 8p11-12 as a site of recurrent translocation and amplification [10,11,14,17,19]. We now present a high-resolution dataset for the whole of 8p with specific follow-up of recurrent events occurring on distal 8p – a relatively unstudied 30 Mb of DNA.

We identified three novel regions of interest on distal 8p: 8p21.3, which contained a novel amplicon centred at 21.8 Mb; 8p22, which had a variety of rearrangements between 12.9 Mb and 15.6 Mb; and a region of consistent loss centred at 1.8 Mb in 8p23.3. These results show that high-resolution BAC array CGH is a good approach for characterising genomic rearrangements to a resolution that provides a manageable number of candidate genes for further study in a cost-effective way.

### Amplification around 21.8 Mb in 8p21.3

A novel amplicon was found in 8p21.3 in both cell lines and primary tumours. Amplification was a relatively rare event, seen in 6% (2/41) of cell lines and 1/98 primary tumours, perhaps due to the reported over-representation of tumours with amplicons in the cell line collection [19]. However, the presence of the amplicon in primary tumour material confirms it as a genuine event in breast cancer. Furthermore, the minimum region of amplification contains candidate genes from families known to play a role in carcinogenesis.

*FGF17 *(*fibroblast growth factor 17*) is a member of the fibroblast growth factor family, a group strongly implicated in carcinogenesis. FGF pathway genes, including *FGF3*, *FGF4 *and *FGF8*, are common targets of MMTV integration [42,43] and *FGFR3 *mutations have been reported in 41% of bladder cancers [44]. *FGF17 *is upregulated in some prostate cancers and this correlates with a higher risk of metastases and lower survival [45]. NPM2 (Nucleophosmin/nucleoplasmin member 2) can catalyse the assembly of DNA and histones into nucleosomes and is involved in decondensation and remodelling of sperm chromatin immediately after fertilisation. In the same family, NPM1 has the ability to regulate the function of the tumour suppressor protein ARF [46], and is mutated in several haematological malignancies including 35% of acute myeloid leukaemias [47,48]. GFRA2 mediates RET signalling in response to the ligand GDNF [49]. RET is a well-known oncogene that plays a role in the development of thyroid carcinomas and the familial cancer syndrome multiple endocrine neoplasia (reviewed in [50]).

Of these genes *FGF17 *and *NPM2 *showed over-expression in two cell lines, one with (MDA-MB-134), and one without (DU4475), amplification. However, neither was over-expressed in the second cell line with amplification in this region (BT-20). If, as the array CGH data suggests, the highest level of amplification in BT-20 lies outside the region amplified in MDA-MB-134, there may be multiple targets of amplification in this region. A similarly complex pattern of non-overlapping amplicon peaks is seen in 8p12 and 11q13 in breast cancer, and has led to the suggestion that they contain as many as four targets [11,51].

### Rearrangements between 12.9 Mb and 15.6 Mb in 8p22

*SGCZ *(14.6 Mb), although disrupted by two unbalanced changes and one balanced translocation breakpoint, is unlikely to be a cancer gene. It forms part of the sarcoglycan complex that links the intracellular cytoskeleton to the extracellular matrix in muscle [52], and our findings show that it is not expressed, either in normal breast or as a consequence of rearrangement. Although *DLC1 *(13.2 Mb) has a tumour suppressive effect in a variety of tumour types, including breast cancer (e.g. [40]), it was not a likely target of these 8p22 rearrangements as it lay just outside the rearrangements in all except one case. This is consistent with suggestions that genomic rearrangement is not a mechanism of *DLC1 *inactivation [39]. If a tumour suppressor gene is present in this region, then *TUSC3 *(15.6 Mb), previously suggested as a potential target in this region ([4,53,54], was the strongest candidate target of the rearrangements of 8p22 in this study. Two cell lines have deletions that solely affect *TUSC3 *and result in decreased expression and expression was lost or decreased in 31% of primary breast tumours. However, the presence of several breakpoints in this region that do not appear to have an effect at the gene level could alternatively suggest that this site is prone to breakage.

### Losses between 1.0 Mb and 2.1 Mb in 8p23.3

Loss of 8p23.3, with a minimum common region of between 1.7 Mb and 1.9 Mb, was found in both breast and pancreatic cell lines, consistent with evidence from LOH studies of a cancer gene situated between 1.0 Mb and 2.1 Mb. 1 Mb array CGH data [55] from primary breast tumours suggests that 8p23.3 is a genuine target in primary tumours. 8% (6/73) of tumours may have specific loss distal to 10 Mb. However, analysis of that dataset is difficult due to the low resolution and the effect of contaminating normal DNA on log_2 _ratio shifts in primary material. In combination, these results suggest the region between 1.0 Mb and 2.1 Mb as the location of a tumour suppressor gene. The genes affected by the minimum common region of deletion, and therefore candidates, are *CLN8*, *C8orf61*, *has-mir-596 *and *ARHGEF10*. *ARHGEF10 *has emerged as a candidate colorectal cancer gene in the sequencing screen carried out by Wood *et al*. [56], and, in conjunction with our discovery of a heterozygous deletion of *ARHGEF10 *in DU4475 with mutation of the remaining copy, this makes it a strong candidate for the 8p23.3 tumour suppressor. Functional data about ARHGEF10 supports a role for it in carcinogenesis. It activates RhoB [57], which is down-regulated in multiple tumour types [58,59] and is necessary for apoptosis in response to DNA damage in transformed cells [60].

### A parsimonious model to explain 8p rearrangements

**Figure 5:**
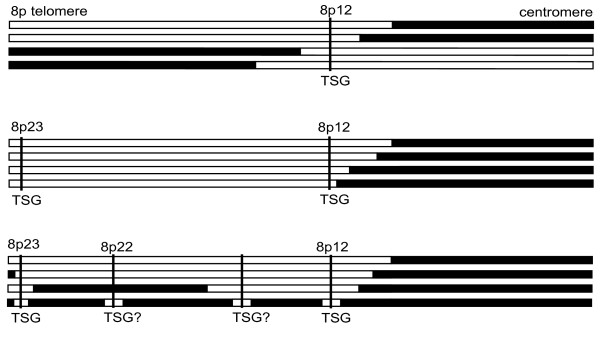
**A parsimonious model of 8p rearrangements**. The different patterns of 8p rearrangement that would be expected in a sample of tumours based on the number and position of tumour suppressor genes (not to scale). Top is the pattern of rearrangements expected if there is a single tumour suppressor gene located in 8p12. Middle is the pattern of rearrangements predicted if there were at least two tumour suppressor genes located on 8p, one in 8p12 and one located close to the telomere in 8p23.3. This fits the common overall pattern of breaks in 8p12 with loss of distal 8p. Bottom is a representation of some of the atypical 8p rearrangements found in cancer cell lines including DU4475, HCC1500 and PMC42. These can be explained by the presence of other tumour suppressor genes in between those in 8p12 and 8p23.3; our results suggest at least one, at 8p22. Since each of these tumour suppressor genes is only lost in a proportion of cases a complex pattern of rearrangements encompassing some or all of these regions results. Loss of the whole of distal 8p will be the most common rearrangement as it removes all tumour suppressor genes as the result of a single event.

A large number of cancers lose all, or nearly all of 8p, potentially suggesting the presence of tumour suppressor loci, while the occurrence of specific rearrangements on distal 8p supports the theory that there are multiple cancer genes located on 8p. Many previous LOH and copy number studies, as well as our data, especially the specificity of the deletions in DU4475, can narrow down the regions of loss to 8p22 and 8p23; we suggest *TUSC3 *and *ARHGEF10 *as possible targets of genomic rearrangement. There may well be other tumour suppressor genes, such as *DLC1*, located on 8p but these appear to be inactivated by alternative mechanisms, including methylation. These results lead us to suggest a parsimonious model to explain the varied rearrangements seen on 8p in cancer (Figure 5)

The most frequent rearrangements are loss of distal 8p, from 8p12 to the telomere, sometimes with proximal amplification of 8p11-12. As previously suggested, unbalanced translocations through 8p11-12 can be explained by the presence of a major tumour suppressor gene at the most distal of these breaks, around 30 Mb [10]. However, we argue that if this were the only tumour suppressor gene on 8p, we might expect to see a mirror-image pattern of breaks distal to this gene with the telomeric sequences being retained, as well as interstitial deletions of the tumour suppressor gene. A tendency towards loss of the whole of distal 8p suggests that there is at least one further tumour suppressor gene, and that it is located close to the telomere (Figure 5). This is consistent with our data suggesting a tumour suppressor gene is located in 8p23.3. There can therefore also be any number of additional tumour suppressor genes in between these two which would give the same pattern of rearrangement, and our data suggests that there may be at least one further candidate gene located in 8p22. In line with the application of Occam's razor loss of both/all of these tumour suppressor genes is most often seen as loss of the whole of distal 8p, but can also be achieved by several more specific events, therefore explaining the complex and inconsistent pattern of changes on distal 8p (Figure 5) – tumours may have deletions of varying sizes encompassing two or more tumour suppressor genes as well as losing them all by loss of the whole of 8p or a proximal translocation.

Cell lines with multiple small deletions are invaluable in allowing us to narrow down the number and position of tumour suppressor genes on 8p. The unique pattern of deletions in DU4475, three of which target candidate tumour suppressor genes or regions previously reported as frequently deleted, supports 8p23.3, 8p22 and 8p21 as candidate regions. Potential targets in 8p23.3 and 8p22 have been discussed above while 8p21, although only affected by a single small deletion in this study, is the location of *RHOBTB2*, a candidate breast cancer gene based on the discovery of two somatic mutations [41], and the TRAIL receptors *DR4 *and *DR5*, which have been suggested as candidate cancer genes owing to their pro-apoptotic function [61]. MIA PaCa-2 has two deletions on 8p, also supporting the existence of tumour suppressor genes at 8p22 and 8p23.3.

Although the premise of our Discussion is that the driving force behind 8p rearrangements is selection for the alteration of cancer genes, the same pattern of rearrangements might be observed if the driving force was the mechanism of breakage e.g the presence of fragile sites on 8p. For example, the breaks at 8p12 with proximal amplification could be due to the presence of a fragile site that initiates breakage-fusion-bridge cycles [62] although no fragile site has been shown to exist on 8p [63] and 8p12 breaks frequently occur without accompanying amplification.

## Conclusion

While early work on 8p, driven by LOH analysis [6,7] focused on distal 8p, more recent studies have concentrated on 8p11-12 [10,11] in an attempt to explain the role of this chromosome arm in cancer. This present study suggests that, in addition to the frequently observed loss from 8p12 to the telomere with proximal amplification, there are additional rarer events that occur on distal 8p. While loss of specific distal loci may occur in only a few cases, loss of these distal sequences may also add to selection for general distal loss following the commonly observed breakage at 8p12 in many tumours.

## Competing interests

The authors declare that they have no competing interests.

## Authors' contributions

SLC wrote the manuscript and carried out most of the experimental work. JP supervised the experimental work. SFC performed tissue microarray experiments under the supervision of CC. IOE produced the tissue microarrays. PAWE is the research group leader and supervised manuscript writing.

## Pre-publication history

The pre-publication history for this paper can be accessed here:



## Supplementary Material

Additional File 1 **Cancer cell lines**. This file contains Table 1 – a list of all cancer cell lines used in the study, culture conditions and source. Click here for file

Additional File 2 **Validation of 8p array CGH**. This file includes the array plot for hybridisation of T47D against normal female on the 8p array platform and FISH validation of the normal copy number polymorphism in 8p23. Click here for file

Additional File 3 **Primers**. This file contains Table 2 – a list of the sequences of all primers used in the study. Click here for file

Additional File 4 **8p array CGH**. This file includes the data for 38 cancer cell lines for which array CGH was performed on the short arm of chromosome 8. Click here for file

Additional File 5 **Copy number changes on 8p**. This file contains Table 3 – a summary of all the copy number changes on 8p identified by array CGH in 38 cancer cell lines. Click here for file

Additional File 6 **Fosmid array CGH**. This file includes the fosmid array CGH data for cell lines with rearrangements in either 8p23.3 or 8p22, between *DLC1 *and *TUSC3*. Click here for file
