# Supplementary material for: High-resolution array CGH clarifies events occurring on 8p in carcinogenesis
Source: BMC Cancer. 2008 Oct 7;8:288. doi: 10.1186/1471-2407-8-288 (PMC2576333; doi:10.1186/1471-2407-8-288)
Supplement: Additional File 1 — Cancer cell lines. This file contains Table 1 – a list of all cancer cell lines used in the study, culture conditions and source. [file 1471-2407-8-288-S1.pdf]

Table 1: Cancer cell lines used

Cell Line	Tissue	Source[3]	Medium[4]	Reference
BT-20	Breast	O'Hare	DMEM/F12	Lasfargues et al, 1958
BT-474	Breast	O'Hare	DMEM/F12	Lasfargues et al, 1978
CaMa-1	Breast	Chaffanet	RPMI-1640	Dobrynin et al, 1963
DU4475	Breast	O'Hare	RPMI-1640	Langlois et al., 1979
HCC1143	Breast	ATCC	See ref	Gazdar et al, 1998
HCC1187	Breast	ATCC	See ref	Gazdar et al, 1998
HCC1419	Breast	ATCC	See ref	Gazdar et al, 1998
HCC1500	Breast	ATCC	See ref	Gazdar et al, 1998
HCC1569	Breast	ATCC	See ref	Gazdar et al, 1998
HCC1599	Breast	ATCC	See ref	Gazdar et al, 1998
HCC1806	Breast	ATCC	See ref	Gazdar et al, 1998
HCC1937[1]	Breast	ATCC	See ref	Gazdar et al, 1998
HCC1954	Breast	ATCC	See ref	Gazdar et al, 1998
HCC38	Breast	ATCC	See ref	Gazdar et al, 1998
HCC70	Breast	ATCC	See ref	Gazdar et al, 1998
HS578t	Breast	O'Hare	DMEM/F12	Hackett et al, 1977
MCF-7	Breast	ATCC	DMEM/F12	Soule, et al. 1973
MDA-MB-134	Breast	O'Hare	DMEM/F12	Cailleau, et al. 1974
MDA-MB-175	Breast	O'Hare	DMEM/F12	Cailleau, et al. 1974
MDA-MB-361	Breast	ATCC	DMEM/F12	Satya-Prakash, et al. 1981
MDA-MB-415	Breast	O'Hare	DMEM/F12	Cailleau et al, 1978
MDA-MB-453	Breast	ATCC	DMEM/F12	Cailleau et al, 1978
MDA-MB-468	Breast	O'Hare	DMEM/F12	Cailleau, et al. 1978
PMC42	Breast	O'Hare	DMEM/F12	Whitehead, et al. 1983
SKBR-3	Breast	ATCC	DMEM/F12	Fogh and Trempe 1975
SUM159	Breast	Orig	See ref	Flanagan et al, 1999
SUM44	Breast	Orig	See ref	Mahacek et al, 1993
SUM52	Breast	Orig	See ref	Ethier et al, 1996
T47D	Breast	ATCC	DMEM/F12	Keydar, et al. 1979
UACC812	Breast	Sanger	DMEM/F12	Meltzer et al, 1991
ZR-75-1	Breast	ATCC	DMEM/F12	Engel et al, 1978
ZR-75-30	Breast	O'Hare	RPMI-1640	Engel et al, 1978
Caov-3	Ovarian	Brenton	DMEM/F12	
PA-1	Ovarian	Brenton	DMEM/F12	Zeuthen et al., 1980
SK-OV-3	Ovarian	O'Hare	McCoy's	Fogh et al, 1977
Capan-1 [2]	Pancreatic	ATCC	RPMI-1640	Fogh et al, 1977
CF-PAC-1	Pancreatic	Lemoine	RPMI-1640	Schoumacher, et al. 1990
MIA PaCa-2	Pancreatic	Lemoine	DMEM/F12	Yunis et al., 1977
PancTu1	Pancreatic	Lemoine	RPMI-1640	Moore, et al. 2001
RWP-1	Pancreatic	Lemoine	RPMI-1640	Dexter, et al. 1982
Suit2	Pancreatic	Lemoine	RPMI-1640	Iwamura, et al. 1987

[1] This cell line is a BRCA1 mutant
[2] This cell line is a BRCA2 mutant

[3] ATCC, American Type Culture Collection, in most cases via the European Collection of Animal Cell Cultures (ECACC, Porton Down, UK)
Brenton, Dr James Brenton, CRUK CRI, Li Ka Shing Centre, Cambridge, UK
Chaffanet, Dr Max Chaffanet (Departement d'Oncologie Moleculaire, Institute Paoli Calmettes, Marseille)
Lemoine, Prof Nicholas R Lemoine (Centre for Molecular Oncology, Institute of Cancer and the Cancer Research UK Clinical Centre, Barts and The London School of Medicine, London, UK)
O'Hare, Prof Michael J. O'Hare (LICR/UCL Breast Cancer Laboratory, University College, Middlesex Medical School, London, UK)
Orig, from the originator
Sanger, from The Sanger Institute, Hinxton, UK
[4] Media abbreviations: DMEM/F12 - Dulbecco's Modified Eagle Media:F12 nutrient mixture, RPMI-1640 - Roswell Park Memorial Institute Medium 1640
