# Supplementary material for: High-resolution array CGH clarifies events occurring on 8p in carcinogenesis
Source: BMC Cancer. 2008 Oct 7;8:288. doi: 10.1186/1471-2407-8-288 (PMC2576333; doi:10.1186/1471-2407-8-288)
Supplement: Additional File 2 — Validation of 8p array CGH. This file includes the array plot for hybridisation of T47D against normal female on the 8p array platform and FISH validation of the normal copy number polymorphism in 8p23. [file 1471-2407-8-288-S2.pdf]

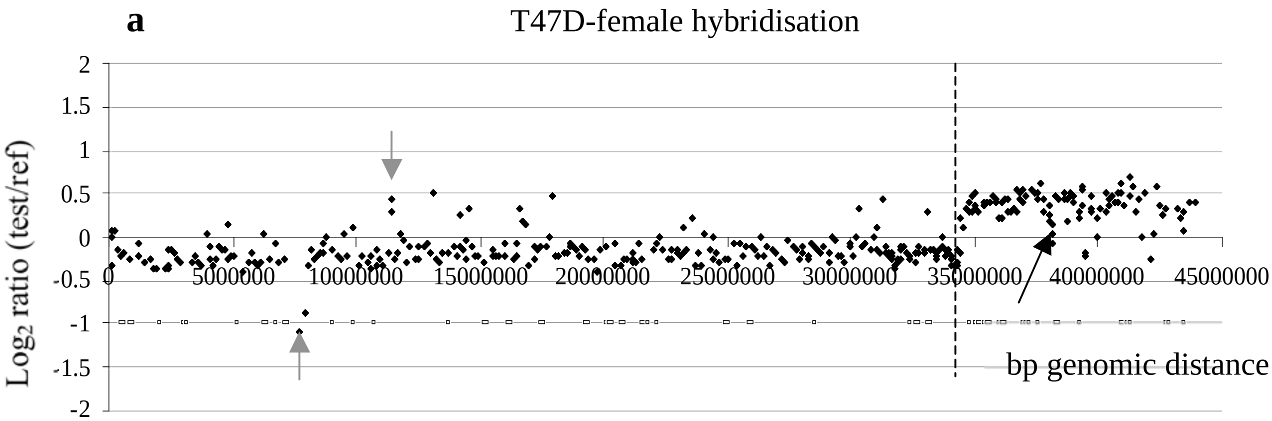

Figure 1a Array CGH for 8p in T47D. The ability of the array to detect copy number variation was evaluated by hybridising it to the T47D breast cancer cell line for which copy number changes on 8p had been extensively characterised by FISH (Pole *et al.*, 2006). A copy number change from two to four in T47D (vertical dashed line) was reflected by a shift in $\log_2$ ratio from -0.18 ± 0.12 S.D. to 0.37 ± 0.14 S.D. In addition, a 110kb deletion of two out of four copies at 38.1Mb (black arrow), a region for which the average $\log_2$ ratio was 0.37, was detected by the array as a single clone with a $\log_2$ ratio of -0.22 and two flanking clones with intermediate $\log_2$ ratios. Black diamonds, hybridisation ratios plotted at BAC midpoints; Open squares, rejected data points; Grey arrows, polymorphism or segmental duplication.

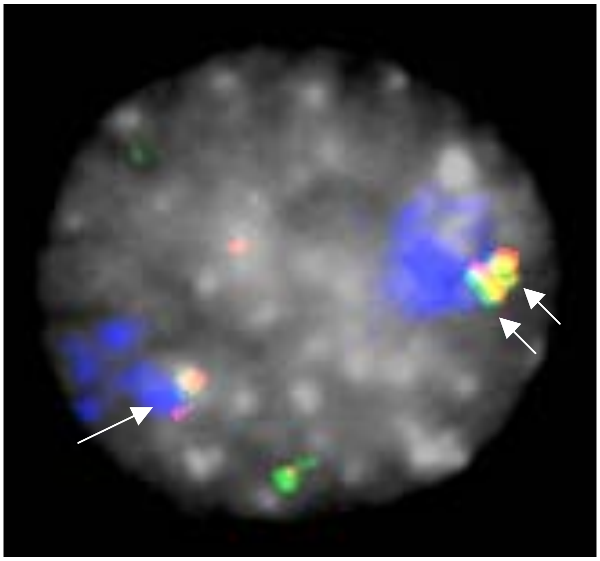

Figure 1b A normal polymorphism in 8p23. A normal lymphoblastoid cell line (m62) interphase nucleus showing a tandem duplication of BAC probes on one copy of chromosome 8 (two arrows) and a single copy of each on the other chromosome 8 (single arrow). Blue, chromosome 8 paint; Red, RP11-43B8 (7.76 Mb); Green, RP11-185K20 (7.80 Mb).
