# Supplementary material for: High-resolution array CGH clarifies events occurring on 8p in carcinogenesis
Source: BMC Cancer. 2008 Oct 7;8:288. doi: 10.1186/1471-2407-8-288 (PMC2576333; doi:10.1186/1471-2407-8-288)
Supplement: Additional File 3 — Primers. This file contains Table 2 – a list of the sequences of all primers used in the study. [file 1471-2407-8-288-S3.pdf]

Primer sequences

EBP49:	5’-GCCAAGATGGACAATCAGGT-3’<br>5’-ATGTGGATTTGGGTGACAGC-3’
FGF17:	5’-ACCAGTACGTGAGGGACCAG-3’<br>5’-CAAACGTGTCCGTCTCCAC-3’
GAPDH:	5’-GCAAATTCCATGGCACCGT-3’<br>5’-TCGCCCCACTTGATTTTGG-3’
GFRA2:	5’-AATTGTCGAGCCTCCTACCA-3’<br>5’-CATGGGTTCTCGGTGAAGTC-3’
NPM2:	5’-CAGTCAGGAGAGGCGGACT-3’<br>5’-TCTTGTCCTCCTGGTTTGCT-3’
TUSC3:	5’- GCTGAGCAACTAGCAAAGTGG-3’<br>5’- CAAATAAAGCAAACCTCCAACA-3’
XPO7:	5’-TGCAACTGCTCAAGCTCACT-3’<br>5’-ATGAAGGAGGGATGGAATGA-3’
