# Supplementary material for: High-resolution array CGH clarifies events occurring on 8p in carcinogenesis
Source: BMC Cancer. 2008 Oct 7;8:288. doi: 10.1186/1471-2407-8-288 (PMC2576333; doi:10.1186/1471-2407-8-288)
Supplement: Additional File 5 — Copy number changes on 8p. This file contains Table 3 – a summary of all the copy number changes on 8p identified by array CGH in 38 cancer cell lines. [file 1471-2407-8-288-S5.pdf]

Table 3: Unbalanced rearrangements of 8p found in 30/41 cancer cell lines by array CGH

Cell Line	Distal BAC	Midpoint[1]	Proximal BAC	Midpoint[1]	Copy no. change[3]
BT20	RP11-419L22	21.14	RP11-582J16	22.50	amplicon
	RP11-322A12	35.38		centromere[2]	increase
CaMa-1	RP11-794M05	17.99	RP11-452M12	18.58	increase
	RP11-380B11	36.92	RP11-762D20	42.33	amplicon
	RP11-749C07	42.47		centromere	increase
DU4475	RP11-784G19	1.06	RP11-439C15	1.85	decrease
	RP11-334K14	5.85	RP11-485I5	6.82	decrease
	RP11-165A17	15.40	RP11-314P10	15.59	decrease
	RP11-67H12	21.94	RP11-364H17	23.00	decrease
	RP11-757J02	27.84	RP11-380I10	28.17	decrease
HCC1143	RP11-331J19	35.90		centromere	increase
HCC1500		telomere	RP11-784G19	1.17	increase
	RP11-632H17	36.50	RP11-701H6	37.99	amplicon
	RP11-389E22	38.14		centromere	increase
HCC1569		telomere	RP11-357H1	3.68	decrease
	RP11-72H22	32.14	RP11-65P16	33.29	increase
	RP11-654O09	34.30		centromere	increase
HCC1599	RP11-203E08	24.03	RP11-701H6	37.99	increase
	RP11-90P05	38.16		centromere	further increase
HCC1806	RP11-683E21	12.62	RP11-68C24	13.61	decrease
	RP11-689I12	14.33	RP11-722M19	31.48	decrease
HCC1937	RP11-719N14	40.07		centromere	increase
HCC1954	CTD-2172C13	39.27		centromere	increase
HCC38	RP11-546K23	3.40	RP11-651E23	17.14	increase
	RP11-608P11	33.10		centromere	increase
MDA-MB-134	RP11-459H21	21.29	RP11-600L04	22.05	amplicon
	RP11-221N11	34.70	RP11-749P01	40.72	amplicon
MDA-MB-175	RP11-431A19	32.28	RP11-405D24	33.24	increase
MDA-MB-361		telomere	RP11-705G10	2.84	increase
	RP11-597D18	34.13	RP11-221N11	34.70	further increase
	RP11-155H15	34.72	RP11-331J19	35.90	increase
	RP11-615C12	36.00		centromere	further increase
MDA-MB-453	RP11-724C15	35.59		centromere	increase
PMC42	RP11-715M14	2.19	RP11-70F13	19.84	increase
SUM44	RP11-68C24	13.61	RP11-50L15	14.63	increase
	RP11-370E21	36.05	RP11-642I24	41.88	amplicon
SUM52	RP11-722M19	31.48		centromere	amplicon
T47D	RP11-369C6	34.67	RP11-583G07	39.31	increase
	RP11-769N08	39.63		centromere	increase
UACC812	RP11-780J19	23.90	RP11-118I24	24.65	increase
	RP11-350N15	38.39	RP11-290A05	42.69	increase
ZR-75-1	RP11-685I22	29.15	RP11-615M04	30.30	increase
	RP11-764B7	31.14	RP11-72H22	32.14	decrease
	10 further breakpoints. See Pole et al. 2006				
ZR-75-30	RP11-723D22	38.82		centromere	increase
CaOV3	RP11-755K01	29.28	RP11-760C14	30.06	increase
PA-1	RP11-45M09	25.88	RP11-722A15	26.23	decrease
Capan-1	RP11-415F24	24.89	RP11-705E05	28.02	increase
	RP11-26K8	37.18	RP11-690P09	38.71	increase
	RP11-104D16	40.25		centromere	increase
CF-PAC-1	RP11-431M3	34.20		centromere	increase
MIA PaCa-2	RP11-796K11	1.76	RP11-439C15	1.85	homozygous deletion

	CTD-2339A14	9.74	RP11-589N15	11.72	homozygous deletion
	RP11-165A17	15.40	RP11-23J14	15.72	increase
	RP11-222M11	19.49		centromere	increase
PancTu1	RP11-212N14	31.58		centromere	increase
RWP-1	RP11-14D16	28.61	RP11-636F12	38.25	increase
	RP11-350N15	38.39		centromere	further increase
Suit2	RP11-158M23	33.19	409I21	34.96	amplification
	79H13	35.03		centromere	increase

[1] Midpoints given are based on NCBI Build 36

[2] Where the rearrangement extends up to or beyond the centromere the most proximal position is given as the centromere.

[3] The copy number change given is relative to the copy number shown for the majority of 8p which may therefore be 1, 2 or more and may or may not reflect the ploidy of the cell line.
